# Representation of Different Types of Adjectival Polysemy in the Mental Lexicon

**DOI:** 10.3389/fpsyg.2021.742064

**Published:** 2021-10-29

**Authors:** Valentina Apresjan, Anastasiya Lopukhina, Maria Zarifyan

**Affiliations:** ^1^School of Linguistics, HSE University, Moscow, Russia; ^2^Vinogradov Russian Language Institute, Moscow, Russia; ^3^Center for Language and Brain, HSE University, Moscow, Russia

**Keywords:** polysemy, adjectives, metonymy, metaphor, mental lexicon, storage, semantic clustering, mental representation

## Abstract

We studied mental representations of literal, metonymically different, and metaphorical senses in Russian adjectives. Previous studies suggested that in polysemous words, metonymic senses, being more sense-related, were stored together with literal senses, whereas more distant metaphorical senses had separate representations. We hypothesized that metonymy may be heterogeneous with respect to its mental storage. “Whole-part” metonymy (“sad person” – “sad eyes”), which is cognitively closer to the literal sense and more regular, should be stored differently from temporal, causal or resultative metonymy (“sad person” – “sad time;” “sad person” – “sad news;” “lead.ADJ ball” – “lead.ADJ poisoning”), which is irregular and semantically distant from the literal sense. We conducted an online experiment with semantic clustering task in which the participants were asked to classify sentences with a literal, proximal metonymic, distal metonymic, or metaphorical sense of an adjective into virtual baskets so that sentences with the same perceived sense were put in the same basket. Our results showed that proximal metonymies were grouped together with the literal sense and with each other more often than with distal metonymies and metaphors. Distal metonymies, in turn, were grouped with literal senses more often than with metaphors. Overall, we concluded that literal senses and proximal metonymies were stored in single representations, distal metonymies formed hybrid representations with literal senses, and metaphors were stored separately from literal senses. Additionally, we discovered that perception of semantic differences is affected by the surrounding senses: distal metonymies were more discernible from literal senses when presented with proximal metonymies, and less so when presented with metaphors.

## Introduction

In theoretical linguistics, polysemy is understood as the widespread phenomenon when words have multiple related senses that arise through processes of semantic change and extension of the literal meaning (such as *birch tree* vs. *genealogical tree*, *free-range chicken* vs. *coconut curry chicken*). There are two main mechanisms of semantic extension: metonymy based on contiguity [*She wore a silver fox* (“fox fur”)] and metaphor based on similarity [*He is an old fox* (“a person sly as a fox”)]. Metonymy is a more regular and predictable extension of the literal sense than metaphor (Apresjan, 1974, 1995; Nunberg, 1979; Lehrer, 1990). Metonymy-based senses are often derived from literal senses via sense-derivation rules (Nunberg, 1979; Pustejovsky, 1995) which apply to whole groups of semantically similar words. For example, words denoting textual objects display regular object/content metonymy: *shredded paper “*object” - *innovative paper “*content,” *torn book* “object” - *interesting book* “content”; words denoting containers display regular container/contents metonymy: *a crystal glass “*container” - *a glass of water* “contents of the container,” *a cast-iron pot* “container” - *a pot of soup “*contents of the container.”

Many experimental studies of both types of polysemy demonstrated that because of their greater relatedness to the literal sense, metonymy-based senses exhibited evidence of being stored together with the literal sense in the mental lexicon, in contrast to less related metaphors that were stored separately (Klepousniotou and Baum, 2007; MacGregor et al., 2015; Lopukhina et al., 2018). However, the existing experimental studies on polysemy do not reflect the complexity of this phenomenon in its entirety. First, they mainly concentrate on nouns and sometimes on verbs, while leaving aside adjectival polysemy, which, with the few exceptions (Lopukhina et al., 2018; Weiland-Breckle and Schumacher, 2018) has not received experimental attention. Yet adjectives are an important and linguistically well-represented category among content words, with unique patterns of polysemy. Second, previous studies consider only coarse-grained distinctions between metonymy and metaphor. However, each of these two semantic extensions has more fine-grained subtypes which might conceivably be stored differently in the mental lexicon. Our research aims at partially bridging this gap.

We consider two types of Russian adjectival metonymy, which we dub “proximal” metonymy and “distal” metonymy. Proximal and distal metonymies are each represented by several metonymic extensions previously described in theoretical studies of polysemy (Apresjan, 1974, 1995; Sandakova, 2010, 2015). Proximal metonymy extends denotations of human emotional states to human body parts or actions: *sad person* - *sad eyes* (“eyes of a sad person”), *smart boy - smart behavior* (“behavior of a smart person”). Distal metonymy extends denotations of emotional and physical states to events that cause these states or to time periods: *sad person - sad news* (“news causing one to feel sad”), *sad person - sad time* (“time during which one feels sad”). Senses based on proximal metonymy contain no added semantic components as compared to literal senses, they merely express relation, and their nominal collocates are semantically related to the nominal collocates of literal senses (humans vs. human appearance and actions). Senses based on distal metonymy contain added components of causality, resultativity or temporality and co-occur with nouns of unrelated semantic classes (humans vs. events and time periods). We consider proximal and distal metonymy against the backdrop of metaphorical senses. We hypothesize that proximal metonymy and distal metonymy will display different patterns in the mental lexicon: distal metonymy might occupy an intermediate position between proximal metonymy and metaphor.

### Theoretical Studies of Metonymy and Metaphor

A large body of theoretical studies has considered metonymy (contiguity) and metaphor (similarity) as two fundamental cognitive principles employed in the human conceptualization of the world (Freud, 1900; Frazer, 1923; Lacan, 1957; Lakoff and Johnson, 1980; Jakobson and Halle, 1956). Many linguistic studies juxtapose metonymy and metaphor as two different strategies of semantic extension (Apresjan, 1974, 1995; Lakoff and Johnson, 1980; Fass, 1997; Panther and Radden, 1999; Radden, 2000; Croft, 2003; Kustova, 2004; Paducheva, 2004; Warren, 2009). Metonymy is viewed as a more regular and semantically less drastic shift than metaphor (Apresjan, 1974, 1995; Nunberg, 1979; Croft, 2003; Warren, 2009). Specifically, metonymy is commonly defined as mapping within the same conceptual domain (Radden and Kövecses, 1999), or an extension in focus from one situation participant to another (Kustova, 2004; Paducheva, 2004). Metaphor is defined as mapping across conceptual domains (Lakoff and Johnson, 1980). For example, the literal sense of the adjective *severe* refers to certain personality traits: *Our school principal is severe to students who are late for class*. The metonymic sense of *severe* defines the appearance or actions of a severe person (*severe look, severe punishment*) remaining within the same conceptual domain of humans. The metaphorical sense of *severe* describes natural phenomena (*severe frosts, severe thunderstorms*), which constitute an entirely different conceptual domain.

However, some theoretical scholars contend that metaphor and metonymy do not always represent two opposing poles. Metonymy and metaphor are not homogeneous and may manifest in intermediate phenomena combining both shifts (Kövecses, 1986, 1990, 2013; Goossens, 1990, 2003; Barcelona, 2003; Dirven, 2003; Taylor, 2003; Ruiz de Mendoza Ibáñez and Galera Masegosa, 2011; Qian, 2016), such as metaphtonymy (*to beat one’s breast, to bite one’s tongue*). Kövecses (1986; 1990; 2013) argues that many emotion metaphors are metonymy-based. For example, the metaphor SADNESS IS DOWN (*He is in low spirits*) is based on the metonymical shift DOWNWARD BODILY ORIENTATION FOR SADNESS, motivated by the actual behavioral response associated with this emotion (drooping body posture, mouth turned down). Barcelona (2003) goes even further, suggesting that possibly all conceptual metaphors are metonymy-motivated, i.e., are generalizations of the actually existing cognitive-experiential links.

Within metonymical and metaphoric domains, there is no homogeneity, either. For example, there is a crucial difference between referential metonymy (Warren, 2006), where an entity stands for another, such as *I read Shakespeare* (“Shakespeare” = “Shakespeare’s works”) and logical metonymy (Pustejovsky, 1995), where an entity stands for a “covert event,” such as *Jack Kerouac began the book around 1949 in New York* (“the book” = “writing the book”).

In addition, within referential metonymy, there are different metonymic subtypes of varying relatedness to the literal sense (Apresjan, 1974, 1995; Lakoff and Johnson, 1980; Radden and Kövecses, 1999; Kövecses, 2002; Panther and Thornburg, 2004; Peirsman and Geeraerts, 2006). For example, so-called linear metonymy (Dirven, 2003), i.e., part-for-whole (*Brussels seeks to boost the industry* in reference to the European Union) and whole-for-part (*Germany won* in reference to a sports team) represents an extension that is very close to the literal sense. On the other hand, conjunctive metonymy (*tea* in reference to a meal), or figurative metonymy (*He’s got a good head on him*) manifest greater extensions of meaning. Likewise, so-called actantial metonymy, e.g., action-for-agent (*He hired the best criminal defense*), action-for-result (*He filed his defense late*), or action-for-instrument (*I need to buy a new reader*) is also a major shift from the literal meaning (Apresjan, 1974, 1995; Radden and Kövecses, 1999).

Although metonymy is best-researched with respect to nouns (Apresjan, 1974, 1995; Lakoff and Johnson, 1980; Pustejovsky, 1995), there is considerable evidence of semantically diverse adjectival metonymy in different languages (Dirven, 1999; Goossens, 1999; Radden and Kövecses, 1999; Seto, 1999; Vosshagen, 1999; Rakhilina et al., 2009; Reznikova et al., 2012; Reznikova et al., 2013; Anashkina and Ivanova, 2016). Adjectival metonymy also varies semantically and regularity-wise (Apresjan, 1974, 1995; Birih, 1995; Sandakova, 2010, 2015). For example, a shift from an attribute of a person to an attribute of a person’s appearance (*grustnyj chelovek - grustnye glaza “*sad person” – “sad eyes”) is minor and rarely acknowledged as a separate sense in dictionaries due to its regular and predictable nature (Sandakova, 2010, 2015). However, a shift from an attribute of a person to an attribute of a time period is more noticeable and less regular: *grustnyj chelovek - grustnoe vremja* “sad person” – “sad time,” but not serdityj chelovek -*serditoe vremya “angry person” – “^∗^angry time.”

Overall, theoretical studies suggest that polysemous adjectives may have metonymic extensions with varying degrees of regularity and semantic relatedness to the literal sense. However, there are currently no experimental studies that explore different types of adjectival metonymy as potential contenders for different cognitive processing and storage. Yet it seems reasonable to surmise that different semantic types of metonymy in adjectives might exhibit different properties vis-a-vis their storage in the mental lexicon.

### Experimental Studies of Metonymy and Metaphor

Many psycholinguistic discussions on polysemy revolve around the storage of word senses in the mental lexicon. The key question in the debate is whether senses of polysemous words are represented as a single entry or as separate entries for separate senses. The proponents of a single-sense representation argue that there is one core representation of each polysemous word in the mental lexicon. The advocates of separate storage contend that different senses of a polysemous word (polysemes) are stored in different mental representations. In between are hybrid storage approaches, which routinely distinguish between different sense types in polysemous words. Hybrid storage adherents suggest that senses that are highly related to the literal sense are stored together with it, whereas less related senses are stored separately.

#### Single-Sense Storage Approaches

Single-sense storage experimental studies corroborate single-sense theoretical models of polysemy which are based on sense-generation or sense-derivation rules, such as Nunberg (1979) or Pustejovsky (1995). Nunberg (1979) suggested a pragmatic single-sense account of regular polysemy, in which different non-metaphorical word uses were explained by lexical conventions, and their referents were established based on context, as in *The book weighed five pounds* (physical book) vs. *The book was refuted* (book contents). Nunberg also advanced a syntactic argument in favor of the single-entry model, based on the fact that regularly derived senses behaved as one item with respect to pronominalization (replacement by a pronoun) and deletion (removal of a piece of syntactic structure in certain conditions) (Nunberg, 1979): *John’s dissertation, which weighs five pounds* (physical dissertation), *has been refuted* (dissertation content). Pustejovsky (1995), likewise, argued for single-sense representation of regular polysemy, where different word uses were derived via lexical rules. A similar theoretical account was suggested in Copestake and Briscoe (1995), where new senses were considered derived through a regular process of *sense extension*.

Experimental single-sense accounts, in a similar vein, claim that senses of a polysemous word “belong to or depend on a single representation” (Falkum and Vicente, 2015). According to the underspecification hypothesis, when encountering a polysemous word, instead of accessing a specific sense, language users initially activate a word’s meaning that is semantically underspecified (Frisson and Pickering, 1999; Frisson, 2009). While homonyms (words having unrelated senses due to historical coincidence) are hypothesized to be stored separately, polysemous words are thought to represent one single entry due to the overlap among their senses (Beretta et al., 2005; Tamminen et al., 2006). The claim is based on the findings of visual (Rodd et al., 2002; Beretta et al., 2005; Frisson, 2009) and auditory (Tamminen et al., 2006) lexical decision experiments that measured access times of homonymous vs. polysemous meanings. Homonyms (such as *bark*, which refers to a tree part or to the sound made by dogs) showed slower access times than polysemous words (such as *belt*, which refers to an article of clothing or a part of machinery). The advantage of polysemy is supposedly caused by a larger shared activation space, while homonymy disadvantage is due to smaller individual activation spaces.

#### Separate Sense Storage Approaches

Single-sense view of polysemy representation is challenged in separate storage approaches. Theoretically, the proponents of separate representation of polysemes have argued that polysemous senses are too unpredictable to be fully derived by linguistic rules (Cruse, 1986; Lehrer, 1990; Rice, 1992). According to the experimental data that support this view, polysemes have separate entries: for example, *paper* as writing material (*shredded paper*) and *paper* as a news periodical (*daily paper*) would possess distinct representations in the mental lexicon, connected to the same lemma.

In a series of behavioral experiments, Klein and Murphy (2001) compared recall rates, judgment correctness, and response speed for phrases with polysemes, primed with consistent or inconsistent word uses, e.g., *liberal paper* vs. *daily paper* (consistent), *wrapping paper* vs. *daily paper* (inconsistent). The results demonstrated advantage for contexts evoking the same senses of polysemous words and disadvantage for contexts evoking different senses. The authors argued that if the words were indeed interpreted in terms of a common core meaning, then memory, judgment and speed would have been equivalent for the consistent and inconsistent senses. Therefore, Klein and Murphy concluded that polysemous words had separate representations for each sense and that any core meaning was minimal. Klein and Murphy (2002) lended further support to the separate sense representation model using experimental evidence in categorization tasks, such as deciding which capitalized word in the choice phrase went better to form a category with the capitalized word in the target phrases (e.g., *wrapping PAPER* and *shredded PAPER* or *liberal PAPER*). They demonstrated that respondents tended to choose either words used in the same sense, or words that are taxonomically or thematically related, but not words used in a different sense. Thus, for the target phrase *wrapping PAPER*, the majority of respondents would choose same-sense *shredded PAPER* or taxonomically similar *smooth CLOTH* (also a material) rather than the different sense *liberal PAPER*.

A similar result was reported in a MEG study by Pylkkänen et al. (2006). Based on an investigation of priming between senses in a sensicality judgment task and magnetoencephalography measures, Pylkkänen et al. (2006) found that sense-related stimuli (such as *lined paper - liberal paper*) showed an effect different from both homonyms (such as *savings bank - river bank*) and semantically related but morphologically and phonetically distinct stimuli (*green book - interested novel*). The authors interpreted this result as evidence that in the mental lexicon polysemes connected to the same lexical representation but were listed distinctly.

#### Hybrid Sense Storage Approaches

In a way, single and separate sense storage approaches are reconciled in hybrid sense storage approaches. Many researchers suggest that word senses are heterogeneous with respect to their representation in the mental lexicon: certain non-literal senses are stored together with the literal sense, while others are stored separately. The majority of hybrid sense storage accounts base their claims on different degrees of sense relatedness as manifested in metonymic and metaphorical extensions. Metonymically derived senses are considered as more closely related with the literal sense and are stored together whereas metaphorical senses are further from literal senses and are stored separately (Klepousniotou, 2002; Klepousniotou and Baum, 2007; Klepousniotou et al., 2008, 2012; MacGregor et al., 2015; Lopukhina et al., 2018).

Klepousniotou and Baum (2007), employing a timed lexical decision task, found that metonymically polysemous words (such as *bottle* for container or its content) were processed faster than homonyms, whereas metaphorically polysemous words (such as *star* for celestial body or person) did not show such an advantage. Similarly, Klepousniotou et al. (2008), showed that in a timed sensicality judgment task, words with highly overlapping senses (metonymy) demonstrated reduced effects of context and sense dominance as compared with words with moderately or low overlapping senses (metaphorical polysemy and homonymy). The authors suggested that the comprehension of ambiguous words was mediated by the semantic overlap of alternative senses.

Behavioral experiments that measure clusterization instead of reaction times also support the hybrid account. For example, the results of a semantic clustering experiment on Russian nouns, verbs, and adjectives in Lopukhina et al. (2018) corroborated single-sense storage for more related metonymic senses and separate storage for less related metaphors. The authors also suggested that the mental representation of polysemous words depended, apart from sense-relatedness, on their word class. In nouns and verbs, literal and metonymic senses were stored together, while metaphorical senses were stored separately. However, in adjectives, metonymic senses significantly overlapped with both literal and metaphorical senses.

Several EEG studies also advance hybrid representation of polysemy. Interestingly, EEG results in MacGregor et al. (2015) supported evidence for different storage of homonyms and polysemous words, as do single-sense storage accounts, but also demonstrated differences between metonymic and metaphorical senses within polysemous words. MacGregor et al. (2015) investigated the time-course of meaning activation of homonyms (such as *coach* or *match*), metaphorical polysemes (e.g., *mouth*), and metonymic polysemes (e.g., *rabbit*). In a visual single-word priming delayed lexical decision task, for targets primed by homonyms, no effects survived, while for polysemous primes (metaphorical and metonymic), different degrees of activation of the target were observed. This result indicates different processing and storage patterns for metonymic and metaphorical senses.

In an EEG study of Russian nouns, Yurchenko et al. (2020) reported differences in the processing of metonymic and metaphorical senses. The authors investigated event-related potentials accompanying the participants’ sensicality judgment of phrases with polysemes used in their literal sense. These phrases were preceded by primes with either the same (literal) or different (homonymic, metonymic, or metaphorical) sense. The results demonstrated that ERP responses to phrases preceded by metonymic primes did not differ from the control condition with the literal primes. At the same time, processing phrases with the literal sense preceded by metaphorical primes showed a very limited priming effect. The difference in priming supports the hypothesis of different storage of metonymic and metaphorical senses: the former share a single representation with literal senses in the mental lexicon, while the latter are stored separately. Similar results were found in the study by Weiland-Breckle and Schumacher (2018) for German. The authors concluded that the processing of metaphors was demanding because it involved a cognitively costly mapping between two unrelated domains, whereas the processing of metonymies required mapping processes within a single domain. In general, all the findings align with the general theoretical and experimental trend to consider metaphorical relations as more “distant” from the literal sense than metonymic relations.

Contrary to the majority of results and based on two lexical decision and semantic categorization experiments, Jager and Cleland (2015) found that metonymy-based senses were stored in separate representations, whereas metaphorical senses were stored together with the literal sense. The authors explained these findings by suggesting that the particular instances of metonymy and metaphor, namely, animal-product metonymy (as in *She wore her silver fox*) and animal-person metaphor (as in *He is a sly fox*) represented a counterexample to the generally accepted claim that metonymy is more sense-related to the literal sense than the metaphor. Therefore, the result by Jager and Cleland (2015) may be due to the bias in the stimuli and may indicate that the pattern of sense storage depends on semantic properties of words.

On the whole, we can hypothesize that the major factor that affects the storage of polysemous words in the mental lexicon is sense-relatedness: both metaphor and metonymy can demonstrate stronger or weaker relatedness to the literal sense, depending on their particular type. However, no previous experimental study explicitly targeted different types of the same sense extension, e.g., proximal and distal metonymy, as potential candidates for different representations in the mental lexicon. Most studies either focused on one specific type of metaphor and metonymy, as in Jager and Cleland (2015), or considered different types indiscriminately (e.g., Klepousniotou and Baum, 2007). Proximal and distal metonymic senses are typical for adjectives; however, experimental studies have not considered metaphor and metonymy in adjectives, with the exception of Lopukhina et al. (2018) and Weiland-Breckle and Schumacher (2018).

### The Present Study

The present study aims to fill some of the existing gaps in the experimental study of polysemy and address different types of metonymy in adjectives, as compared to their literal and metaphorical senses. We test the hypothesis that metonymic senses exhibiting strong and weak semantic relatedness to the literal sense, i.e., proximal and distal metonymies, differ with respect to their storage in the mental lexicon, both from each other and from the metaphors.

Proximal metonymy in our study is represented by two regular extensions:

• from an attribute of a person to an attribute of person’s body or appearance: *grustnyj chelovek - grustnye glaza* “sad person” – “sad eyes”; *grustnyj chelovek - grustnyj vzgljad* “sad person” – “sad look;”
• from an attribute of a person to an attribute of a person’s actions: *umnyj chelovek* - *umnoe povedenie “*smart person” – “smart behavior.”

These extensions are the adjectival counterpart of “WHOLE-PART” metonymy (Rakhilina et al., 2009), which is considered a very minor semantic extension from the literal meaning (Lakoff and Johnson, 1980; Dirven, 2003; Panther and Thornburg, 2004; Sandakova, 2010, 2015; Arapinis, 2015).

Distal metonymy in our study is represented by three extensions:

• from a state of a person to time periods during which the state was experienced: *grustnyj chelovek - grustnoe vremya* “sad person” – “sad time;”
• from a state of a person to objects and events causing that state: *grustnyj chelovek - grustnyj pejzazh* “sad person” – “sad landscape”; *grustnyj chelovek - grustnoe sobytie* “sad person” – “sad happening;”
• from a property of a person or object to states resulting from that property: *golodnyj chelovek - golodnyj obmorok “*hungry person” – “hungry fainting,” *saxarnyj sirop - saxarnyj diabet “*sugar.ADJ sirop” – “sugar.ADJ diabetes.”

Compared to proximal metonymy, distal metonymy is less regular and exhibits a far more significant extension from literal senses prompted by the addition of temporal, causal, or resultative semantic components. These shifts are described as subtypes of metonymy in the relevant theoretical studies on adjectival polysemy (Birih, 1995; Kustova, 2004; Paducheva, 2004; Rakhilina et al., 2009; Sandakova, 2010, 2015; Reznikova et al., 2012, 2013). Indeed, they are different from metaphors: they are not based on similarity and, rather than mapping *across* conceptual domains, they represent *“within-domain”* mappings (Barcelona, 2003). All these usages involve referential shifts from one situation participant (Experiencer) to another (Time, Cause, or Result) within the same conceptual domain of human emotions. “Sad time” means time during which the Experiencer is sad in the literal sense; “sad event” means an event that makes the Experiencer sad in the literal sense; “hungry fainting” is fainting resulting from the Experiencer being hungry in the literal sense. In contrast, metaphors, such as *veselyj veter* “cheerful wind” or *sladkaja ulybka* “sweet smile” invoke mapping across conceptual domains. “Cheerful wind” is based on the conceptual metaphor NATURAL PHENOMENA ARE PEOPLE (cf. also *laskovoe solntse* “tender sun,” *grustnye ivy* “sad willows”). *Sladkaja ulybka* “sweet smile” is based on the conceptual metaphor EMOTIONAL IS PHYSICAL or, more specifically, EMOTION IS TASTE (cf. also *gor’kie mysli “*bitter thoughts,” *kisloe nastroenie* “sour mood”). Neither “cheerful” nor “sweet” can be understood literally at any step of semantic decomposition; instead “cheerful wind” is *like* “a cheerful person,” “sweet smile” is *like* “a sweet candy.”

We suggest the following hypotheses regarding the mental representation of different senses in polysemous adjectives: (1) proximal metonymy should have a greater overlap with the literal sense than distal metonymy; (2) distal metonymy should have a greater overlap with the literal sense than metaphor; (3) proximal and distal metonymy should have a greater overlap with each other than any type of metonymy with metaphor. In order to estimate which types of senses in polysemous adjectives overlap and which do not, we apply a semantic clustering paradigm suggested in Lopukhina et al. (2018). We ask participants to cluster short contexts expressing literal, proximal metonymic, distal metonymic, or metaphorical senses of an adjective together into virtual baskets, with an unlimited potential number of categorization groups. We measure how well the participants group together sentences with the same sense and what misclassifications they might have. The semantic clustering approach with multiple options solves both the problem of forced choice and the problem of restricted context, which was typical for previously used categorization paradigms (Klein and Murphy, 2002; Jager and Cleland, 2015). We assume that the offline clustering paradigm implies deep semantic processing of word senses in context because the participants can spend as much time as they need analyzing subtle differences in sense relatedness and deciding about each context. For example, using the offline categorization task Klein and Murphy (2002) showed that the participants sometimes group together different senses of one word (*wrapping paper* and *liberal paper*) that was not detected in the online lexical decision task (Klein and Murphy, 2002). Therefore, we suppose that the clustering task with multiple options provides favorable conditions to investigate semantic representations of polysemous words. In the present study, we expect participants to confuse literal senses with proximal metonymic senses more often than with distal metonymic senses. Distal metonymic senses will be confused with literal senses more often than metaphors. Proximal and distal metonymies will be confused with each other more than with metaphor.

We also investigate how the properties of the experimental paradigm and the way experimental stimuli are presented to participants may influence their decisions about sense grouping. Our stimuli belong to four polysemy types, with three different senses in each type: (1) literal sense with two proximal metonymies (“smart person,” “smart eyes,” “smart behavior”); (2) literal sense with proximal and distal metonymy (“cheerful girl,” “cheerful voice,” “cheerful song”); (3) literal sense with proximal metonymy and metaphor (“cheerful girl,” “cheerful voice,” “cheerful wind”); (4) literal sense with distal metonymy and metaphor (“cheerful girl,” “cheerful song,” “cheerful wind”). We surmise that the perception of semantic differences will be facilitated if the stimuli set contains subtler semantic shifts and impeded if it contains stronger semantic shifts. For example, we expect that distal metonymy (“cheerful song”) will be better identified as separate from the literal sense (“cheerful girl”) when presented together with proximal metonymy (“cheerful voice”), and not as readily when presented together with metaphor (“cheerful wind”). In the first case, the surrounding sentences will behave as “eye-sharpeners,” pushing the participants to search for distinctions between similar items, whereas in the second case, the surrounding sentences will behave as “eye-blinders” — a more distinct sense will divert attention from subtle differences.

The novelty of our research is threefold. In terms of material, we consider polysemous adjectives, which have almost never become an object of experimental study with regard to their representation in the mental lexicon. In terms of granularity, we consider metonymies with varying degrees of relatedness to the literal sense as potential candidates for different representation in the mental lexicon, a never-before-attempted feat in the experimental study of polysemy. Finally, in terms of methodology, we test the hypothesis that semantic clustering of the same sense types may depend on other surrounding sense types.

## Materials and Methods

### Participants

Overall, 1809 adult participants, native speakers of Russian with no self-reported history of neurological and psychiatric disorders, took part in our online experiment. Of these participants, 1153 were recruited via an online platform Toloka^1^ and the other 656 were recruited via social networks. A total of 1485 participants who made no errors in filler trials were selected for further analysis in the experiment. Out of them, 500 participants were male, 984 were female, and one participant was of “other” gender^2^. The age of participants ranged from 18 to 70 years old (M = 34, SD = 11), with 1345 (74.3%) of the participants being right-handed. All participants gave informed consent in accordance with the Declaration of Helsinki.

### Materials^3^

From the Big Explanatory Dictionary of Russian (2014), we selected 39 polysemous adjectives with literal, metonymic, and metaphoric senses. Each adjective had one literal and two non-literal senses, ranging from proximal metonymy to metaphor.

The adjectives for the experiment were selected based on their polysemy structure. We studied four types of polysemy, with the following sense combinations within a single adjective: (1) literal sense, proximal metonymy, proximal metonymy; (2) literal sense, proximal metonymy, distal metonymy; (3) literal sense, proximal metonymy, metaphor; (4) literal sense, distal metonymy, metaphor. Sense types were formulated based on theoretical descriptions. Each stimulus sentence contained a type of shift previously identified by theoretical scholars of adjectival polysemy as metonymical or metaphoric. Our further classification of metonymical senses into proximal and distal metonymy, suggested on semantic grounds, was additionally supported by the dictionary data: metonymical senses which we deemed proximal, were presented within the same subentry with the literal sense (e.g., “smart person” would be numbered as 1.1, and “smart eyes” as 1.2), while metonymical senses which we deemed distal, were presented as separate subentries (e.g., “hungry person” would be numbered as 1, and “hungry years” as 2). Based on theoretical research, we identified the relevant semantic classes of adjectives which were likely to display the sought polysemy structures, and selected specific adjectives as candidates for inclusion in the experiment. Then, using collocational data from the Russian National Corpus, we formulated several short phrases representing each relevant sense of the selected adjectives. Preliminary classification into sense types was performed by each of the three authors, all linguists. For our experiment, we selected the stimuli where the three authors had zero disagreement in the classification.

Proximal metonymy is represented in our data set by the following two extensions described in (Apresjan, 1974; Birih, 1995; Kustova, 1998; Sandakova, 2010, 2015; Apresjan et al., 2014, Apresjan, 2017):

(1.1) from an attribute of a person to an attribute of a person’s appearance: *grustnyj chelovek - grustnye glaza* “sad person” – “sad eyes,” *glupaja maloletka – glupyj smex* “stupid youngster” – “stupid laughter;”
(1.2) from an attribute of a person to an attribute of a person’s actions: *umnyj chelovek* - *umnoe povedenie “*intelligent person” – “intelligent behavior;” *dobrodushnyj djad’ka* – *dobrodushnye shutki “*amiable man” – “amiable jokes.”

The semantic change in the resulting senses compared to the literal sense is minimal: there is no change of domains, as the referents extend from humans to their body parts or actions. The two types of metonymy are widespread among adjectives denoting mental and emotional human properties, such as *bezzabotnyj* ‘‘carefree,’’ *bezumnyj* ‘‘crazy,’’ *blagorodnyj* ‘‘noble,’’ *agressivnyj* ‘‘aggressive,’’ etc., Some adjectives possess both meanings, and some only one of the two. We estimated the regularity of these two extensions in the Russian National Corpus^4^ for a sample of 30 Russian adjectives denoting emotional states. ‘‘Appearance’’ metonymy occurs in 100% of the adjectives in the sample, and ‘‘action’’ metonymy in 83% (see corpus regularity data in the online repository^5^).

Distal metonymy in our sample comprises the following extensions:

(2.1) from a state of a person to time periods *during* which the state was experienced: *grustnyj chelovek - grustnoje vremya* “sad person” – “sad time;” *bezzabotnyj rebenok – bezzabotnye dni* “carefree child” – “carefree days;”
(2.2) from a state of a person to objects and events *causing* that state: *grustnyj chelovek - grustnyj pejzazh* “sad person” – “sad landscape;” *grustnyj chelovek - grustnoje sobytie* “sad person” – “sad happening;” *nervnye deti – nervnaja situatsija* “nervous children” – “nervous situation” (“nerve-wracking”);
(2.3) from a property of a person or object to states *resulting* from that property: *golodnyj chelovek - golodnyj obmorok “*hungry person” – “hunger fainting,” *solnechnyj svet - solnechnaja allergija “*solar light” – “solar allergy;” *svinstovyj sharik – svintsovoje otravlenie* ‘leaden ball” – “lead poisoning.”

In distal metonymy, the sense-relatedness with the literal sense is weaker because of important semantic additions — temporality in the first extension, causality in the second and resultativity in the third. Distal metonymy is also considerably less regular than proximal metonymy. Extensions (2.1), (2.2) are found, respectively, in 23 and 20% of Russian adjectives denoting emotional states in our dataset of 30 words. The chi-square goodness-of-fit tests showed that the occurrence of proximal vs. distal metonymy types was significantly different in the adjectives from the sample χ2 (3, *N* = 68) = 26.706, *p* < 0.001. To identify the direction of difference, we ran additional *post hoc* tests with multiple pairwise comparisons. We found that the person-to-appearance extension of proximal metonymy occurs significantly more often than the temporal (*p* < 0.001) and causal (*p* < 0.001) extensions of distal metonymy. The same pattern was observed for the person-to-action extension of proximal metonymy: it occurs in adjectives significantly more often than the temporal (*p* = 0.002) and causal (*p* = 0.001) extensions. At the same time no significant differences in occurrence were observed between the two different extensions within either the proximal (*p* = 0.6) or distal (*p* = 0.78) types of metonymy.

The extension (2.3) is limited to individual (mostly relative), adjectives that are used with nouns denoting adverse physical states and diseases (e.g., “drowsy potion,” “dead water,” “milk.ADJ allergy,” “sugar.ADJ diabetes,” etc.).

Metaphors included several well-known subtypes of personification, anthropomorphism, and abstraction shifts (Apresjan, 1974, 1995; Lakoff and Johnson, 1980; Kövecses, 2010), for example:

(3.1) object is a person: *umnyj chelovek* - *umnyj zamok* “smart person” – “smart lock;”
(3.2) natural phenomenon is a person: *veselaja devochka - veselyj veter* “cheerful girl” – “cheerful wind;”
(3.3) mental is physical: *trezvyj voditel’ - trezvaja otsenka riskov* “sober driver” – “sober assessment of risks;”
(3.4) emotional is physical: *sladkij pirog - sladkaja ulybka* “sweet cake” – “sweet smile;”
(3.5) color is substance: *molochnyj koktejl’ - molochnyj tsvet* “milky cocktail” – “milky color;” and some others.

The 39 adjectives belonged to one of the four types according to the structure of their polysemies. Three types contained 9 adjectives each, and one type contained 10 adjectives. An adjective *veselyj* “merry” appeared in two types.

• **Type 1** had the following senses: literal — proximal metonymy 1 (property-for-appearance) — proximal metonymy 2 (property-for-behavior), such as *glupyj rebenok - glupaja ulybka - glupyj postupok “*stupid child” – “stupid smile” – “stupid action.”
• **Type 2** had the following senses: literal — proximal metonymy (property-for-appearance or property-for-behavior) — distal metonymy (state-for-time or state-for-cause), such as *golodnyj rebenok - golodnyj vid - golodnye gody* “hungry child” – “hungry look” – “hungry years;” *ljubopytnyj uchenik- ljubopytnye glaza - ljubopytnaja stat’ja* “curious pupil” – “curious eyes” – “curious paper.”
• **Type 3** had the following senses: literal — proximal metonymy (property-for-appearance or property-for-behavior) — metaphor, such as *strogij direktor - strogij ton - strogoe zdanie* “stern/strict principal” – “stern voice” – “stern/austere building.”
• **Type 4** had the following senses: literal — distal metonymy (state-for-cause or property-for-result) — metaphor, such as *veselye tovarishchi - veselaja muzyka - veselye ruchejki* “merry/happy comrades” – “merry/jaunty music” – “merry/vivid stream;” *solnechnyj svet - solnechnaja allergija - solnechnaja ulybka* “solar light” – “solar allergy” (solar eczema) – “solar smile” (sunny smile, radiant smile).

Each sense of each adjective was represented by one, two, or three short sentences featuring adjectives in attributive function. In the sentences, adjectives formed noun phrases with nouns that belonged to different semantic classes: people (“brave boy,” “stern teacher”), human appearance and overt manifestations (“brave look,” “stern voice”), human actions (“brave protest,” “stern rebuke”), artifacts (“sugary syrup”), natural phenomena (“solar beam”), etc. In Types 1–3, all senses were illustrated with two or more sentences. In Type 4, five distal metonymies and one metaphor were illustrated by one sentence, due to difficulties of creating stimuli satisfying all the semantic conditions. The sentences were designed based on simplified and shortened versions of corpus examples from the Russian National Corpus. The total number of stimuli sentences was 263. The average number of sentences for an experimental adjective was 7.1 (SD = 1.13; range 4–9).

Fillers included homonymous and monosemous adjectives, such as *polovaja zhizn’* “sex.ADJ life” vs. *polovaja plitka* “floor.ADJ tiles” (homonymy); *shirokaja ulitsa* “wide street” vs. *shirokaja krovat’* “wide bed” (monosemy). The total number of filler sentences was 73. The average number of sentences for a filler was 5.6 (SD = 1.71; range 3–9). All stimuli and filler sentences are available online (see footnote 3). Sample sentences from each stimuli type are presented in Table 1.

**TABLE 1:** Sample sentences for each of the four types of polysemy (one sentence per sense).

Polysemy Type	Sense Type	Stimulus Sentence	Translation
1	literal	Ответы на вопросы внимательный читатель сможет найти на ϕоруме.	An attentive reader will be able to find answers to questions on a forum.
1	proximal metonymy 1	Это был маленький старичок с умными, внимательными глазами	It was a little old man with smart, attentive eyes.
1	proximal metonymy 2	При внимательном изучении снимков можно узнать некоторые интересные ϕакты.	By attentive study of the images, you can learn some interesting facts.
2	literal	За дверью гавкает голодная собака.	A hungry dog barks outside the door.
2	proximal metonymy	Вокруг нас вились босоногие дети с голодными глазами.	Barefoot children with hungry eyes hung around us.
2	distal metonymy	Однажды с Кузьмой случился голодный обморок.	One day Kuzma had a hungry swoon.
3	literal	У меня в оϕисе очень злой начальник.	I have a very evil boss in the office.
3	proximal metonymy	У него были маленькие злые глазки.	He had small evil eyes.
3	metaphor	Вокруг них росла злая крапива.	Evil nettles grew around them.
4	literal	Посреди шумного общества, в кругу веселых товарищей, ему было хорошо.	In the midst of noisy society, in the company of merry comrades, he was well.
4	distal metonymy	Под веселую музыку в парке танцевали люди.	People danced to the merry music in the park.
4	metaphor	Поблескивая на солнце водяными бликами, журчали веселые ручейки.	Merry streams gurgled in the sun with glittering water.

*We provide literal translations of the adjectives in sentences which are sometimes not felicitous in English, since not all Russian metonymies and metaphors are directly translatable into English.*

### Procedure

**FIGURE 1:**
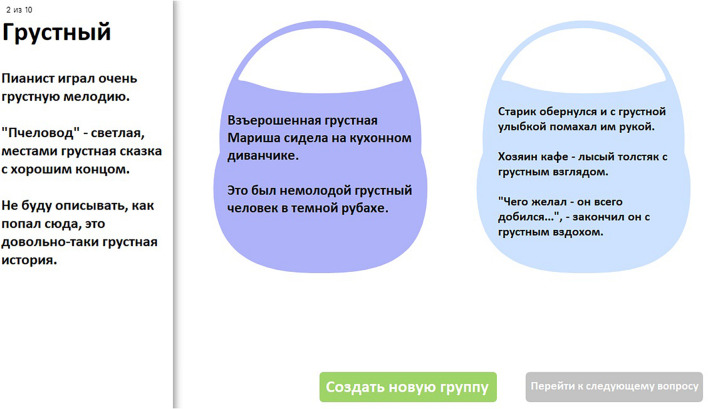
An example trial with the word *grustnyj* (“sad”). In the left column, all phrases that should be sorted are listed. Below are the two buttons “Create a new group” and “Switch to the next trial.” Participants were not allowed to switch to the next trial until they finished sorting all the phrases from the left column.

We used the basket paradigm suggested in Lopukhina et al. (2018). This paradigm is an online questionnaire^6^ in which the participants were asked to sort sentences containing the same adjective so that the sentences with the same perceived adjective sense should be dragged and dropped into the same virtual basket (see Figure 1). The participants could create as many baskets as there were sentences in the list. Trials corresponding to different adjectives were presented in random order, but the order and the number of the sentences within the trial was the same for each participant. The paradigm was programmed in Python and JavaScript, the code is available online: https://github.com/lopuhin/sense-grouping-survey.

Each participant saw 10 different adjectives (7 experimental and 3 filler), and from 45 to 84 stimuli sentences (mean = 66.5) in total. The stimuli adjectives were randomized in such a way that each stimulus was presented to 257 participants on average, ranging from 235 to 292 participants (*M* = 257.13, SD = 14.11).

### Analysis

The raw data files containing the output of the online experiment were preprocessed and annotated with Python for the further analysis. The Python script and the raw data files are stored in the repository together with other materials https://github.com/MariaZarifyan/Representation-of-different-types-of-adjectival-polysemy-in-the-mental-lexicon.

In each of the four Types, we analyzed both correct classifications and misclassifications. By correct, we mean classifications that involved placing all sentences with one sense in the same basket separately from sentences with other senses, similarly to Lopukhina et al. (2018). Misclassifications were defined as deviations from correct classification. For correct classifications, we used a chi-squared test in R (R Core Team, 2016) to evaluate which senses were classified better within each of four Types. We counted the total number of baskets that contained the sentences with one sense within each Type separately (e.g., number of baskets that contained sentences with only literal senses, only proximal metonymies, and only distal metonymies within the Type 2). As a null hypothesis (H0), we assumed that all three senses within each Type should be classified equally correctly. According to alternative hypothesis (H1), some senses might be classified correctly significantly more often than others within each Type.

For misclassifications, we measured the pairwise similarity between data clusterings of senses using the Adjusted Rand Index (ARI; Hubert and Arabie, 1985; Pedregosa et al., 2011). ARI is typically used in statistics and machine learning to estimate how well a set of objects is divided into groups. In our study, for each classification of contexts provided by each participant, we calculated how similar this classification was to the reference expert classification, taking into account two compared senses (e.g., literal sense and metaphor or literal and distal metonymy). Then we averaged the measures of the quality of clusterization and applied bootstrapping by participant to estimate the confidence intervals and whether the differences between Types were significant. ARI corrects for an unbalanced number of contexts for each stimulus, while averaging accounts for the different number of participants who saw each stimulus. Therefore, Adjusted Rand Index allowed us to compare the quality of clusterization between Types.

## Results

First, we analyzed correct classifications of senses. We considered all baskets with correct classifications in each of the four Types as 100%. If the participants classified sentences strictly according to the senses of the adjectives, then the percentages of baskets with correct classifications will be the same for each of the three senses in each of the Types (e.g., baskets with literal senses, proximal metonymic senses and distal metonymic senses in Type 2 will constitute about 33% each). If the participants classified some senses better than the others, then the percent for each of the three senses in the Type will be uneven. The chi-square goodness-of-fit tests showed that within each Type the percent of baskets with correct classifications was uneven between senses: Type 1, χ2 (2, *N* = 2537) = 159.83, *p* < 0.001; Type 2, χ2 (2, *N* = 2533) = 32.253, *p* < 0.001; Type 3, χ2 (2, *N* = 3210) = 459.09, *p* < 0.001; Type 4, χ2 (2, *N* = 3718) = 201.84, *p* < 0.001, see Table 2. To identify the direction of difference, we ran additional *post hoc* tests with multiple pairwise comparisons. We applied a Bonferroni correction for the total number of fitted models, so that the predictors are significant at the α = 0.004. Below we reported uncorrected *p*-values.

**TABLE 2:** Proportion of correct classification for each of the three senses in each Type (each Type = 100%).

Type 1		Type 2		Type 3		Type 4	
literal	45%	distal metonymy	37.8%	metaphor	50.6%	metaphor	43%
proximal metonymy 2	29.1%	literal	33.6%	literal	28.7%	literal	33%
proximal metonymy 1	25.9%	proximal metonymy	28.6%	proximal metonymy	20.7%	distal metonymy	24%

We found that in Type 1, literal senses were placed in separate baskets significantly more often than either of the two proximal metonymies (*p* < 0.001). Proximal metonymy 2 was placed in separate baskets more often than proximal metonymy 1, although this difference was non-significant after Bonferroni correction (*p* = 0.026). In Type 2, distal metonymies were placed in separate baskets more often than literal senses, although the difference was non-significant (*p* = 0.014), whereas literal senses were placed separately significantly more often than proximal metonymies (*p* = 0.002). Sense classification in Types 3 and 4 was similar: metaphors were placed in separate baskets significantly more often than literal senses (*p* < 0.001), and literal senses were placed separately significantly more often than metonymies (*p* < 0.001).

Second, we analyzed the misclassifications made by the participants, see Table 3 for the proportions of incorrect classifications within each Type and Table 4 for the Adjusted Rand Index information. We found that most often the participants incorrectly placed two proximal metonymies in one basket in Type 1 (ARI = 0.292, lower ARI indicates worse discrimination of senses). Least often the participants misgrouped literal senses and metaphors in Type 3 (ARI = 0.835, higher ARI indicates better discrimination of senses) and Type 4 (ARI = 0.812). The participants also rarely confused metonymies and metaphors: ARI for proximal metonymy and metaphor was 0.636 and for distal metonymy and metaphor 0.671, this difference was significant (*p* = 0.008). Finally, for the Type 2, where we could compare the misclassifications of literal senses, proximal metonymies and distal metonymies, we found that literal senses were grouped with proximal metonymies more often than with distal metonymies (ARIs were 0.447 and 0.574, respectively, *p* < 0.001). Within the same Type, proximal and distal metonymies were misclassified together most often (ARI = 0.423, *p* = 0.03 for literal and proximal metonymy, *p* < 0.001 for literal and distal metonymy).

**TABLE 3:** Proportion of misclassifications (each Type = 100%).

Type 1. Literal, proximal metonymy 1, proximal metonymy 2	proximal metonymy 1 + proximal metonymy 2	45%
	literal + proximal metonymy 1	21%
	literal + proximal metonymy 1 + proximal metonymy 2	20%
	literal + proximal metonymy 2	14%

Type 2. Literal, proximal metonymy, distal metonymy	proximal metonymy + distal metonymy	32%
	literal + proximal metonymy	31%
	literal + proximal metonymy + distal metonymy	21%
	literal + distal metonymy	16%

Type 3. Literal, proximal metonymy, metaphor	literal + proximal metonymy	62%
	proximal metonymy + metaphor	27%
	literal + proximal metonymy + metaphor	7%
	literal + metaphor	4%

Type 4. Literal, distal metonymy, metaphor	literal + distal metonymy	56%
	distal metonymy + metaphor	30%
	literal + metaphor	8%
	literal + proximal metonymy + metaphor	6%

**TABLE 4:** Adjusted Rand Index (with standard deviation) for each pairwise comparison.

	**Type 1**	**Type 2**	**Type 3**	**Type 4**
literal + proximal metonymy	1: 0.514 (0.01) 2: 0.577 (0.01)	0.447 (0.01)	0.400 (0.01)	na
literal + distal metonymy	na	0.574 (0.01)	na	0.489 (0.01)
literal + metaphor	na	na	0.835 (0.01)	0.812 (0.01)
proximal metonymy 1 + proximal metonymy 2	0.292 (0.01)	na	na	na
proximal metonymy + distal metonymy	na	0.423 (0.01)	na	na
proximal metonymy + metaphor	na	na	0.636 (0.01)	na
distal metonymy + metaphor	na	na	na	0.671 (0.01)

*Higher ARI indicates better discrimination of the two senses. ARIs can be compared between Types.*

Third, to evaluate the hypothesis that the classification of the same senses (literal or distal metonymies) may depend on stimuli surroundings, we compared the patterns of misclassifications of the same pairs of senses between Types. We found the eye-sharpener effect for the literal sense and proximal metonymy in Type 1 (ARIs 0.514 and 0.577) compared to the eye-blinder effect in Type 2 (ARI = 0.447) and 3 (ARI = 0.400), the difference between all ARIs was significant (*p* < 0.002 or less). For the distal metonymy and literal sense, we also found the eye-sharpener effect in Type 2 (ARI = 0.574) compared to the eye-blinder effect in Type 4 (ARI = 0.489), *p* < 0.0001. Overall, the results of the comparison of misclassifications between Types indicate that stimuli surroundings indeed influence the perception of semantic closeness and provoke the participants to group the same types of senses together less often in the presence of a more distinct sense (eye-blinder effect) and more often in the presence of a less distinct sense (eye-sharpener effect).

## Discussion

The goal of the present study was to test whether proximal metonymic, distal metonymic, and metaphorical senses of Russian adjectives differ with respect to their storage in the mental lexicon. Additionally, we wanted to estimate whether the speakers’ perception of semantic difference between two senses depended on the other surrounding senses. We ran a semantic clustering experiment in which participants were asked to sort sentences with literal, proximal (whole-part) metonymic, distal (causal, resultative, and temporal) metonymic, and metaphorical senses of a word. Our first finding is that senses indeed form a hierarchy with regard to their mental representations as reflected in the speakers’ judgment, depending on their relatedness to the literal sense. Proximal metonymies were grouped together with the literal sense and with each other more often than distal metonymies and metaphors. This demonstrates that language speakers are most sensitive to the distinctions between literal senses and metaphors, which is consistent with their separate representation in the mental lexicon. Distal metonymies are less identifiable as distinct senses, which might point to a greater overlap with literal senses in the mental representation. Finally, proximal metonymies were judged as distinct senses least often, which may indicate their greatest overlap with the literal senses. Our second finding is that although metaphors were very rarely confused with other senses, when they did, they were confused with metonymies considerably more frequently than with the literal senses. Finally, our third finding is that the perception of senses as distinct was affected by their presentation in the experiment: presentation of distal metonymy together with proximal metonymy facilitated its recognition as separate from the literal sense, whereas its presentation together with metaphor prompted its confusion with the literal sense.

The first finding that the mental representation of non-literal senses of a polysemous adjective depends on their relatedness to the literal sense is consistent with the hybrid sense storage approaches (Klepousniotou, 2002; Klepousniotou and Baum, 2007; Klepousniotou et al., 2008, 2012; MacGregor et al., 2015; Lopukhina et al., 2018). However, we advanced further, as we examined not only different semantic extensions (metonymy and metaphor), but also fine-grained semantic distinctions within metonymy, previously described in theoretical studies (Apresjan, 1974, 1995; Birih, 1995; Kustova, 1998; Sandakova, 2010, 2015) yet never before tested experimentally. The general tendency for closely related senses to overlap with the literal sense and for unrelated senses to be perceived as separate, demonstrated in previous experimental studies, is relevant for different subtypes of the same semantic extension as well. Interestingly, we also discovered differences between the two proximal metonymies: proximal metonymy “human property – human action” (“stupid behavior”) was confused with the literal sense less often than proximal metonymy “human property - human body part” (“stupid eyes”). Although both metonymies are close to the literal sense, “human property - human action” metonymy seems to be on a greater semantic distance from the literal sense than “human property - human body part” metonymy. As Lopukhina et al. (2018) suggest, polysemy seems to be a continuum, and speakers appear sensitive to different degrees of relatedness in semantic extensions.

On a polysemy continuum, distal metonymies that represent a greater semantic leap from the literal sense, as compared to proximal metonymy, were more often classified correctly than the latter (e.g., 37.8% of correct classifications for distal and 28.6% for proximal metonymies in Type 2). Metaphors are by far the most “drastic” of semantic extensions, and therefore, in our experiment, speakers rarely confused them with literal senses (ARI of 0.835 in Type 3 and 0.812 in Type 2, the highest for all possible misclassifications). However, distal metonymy was classified with the literal sense more often than metaphor (ARI of 0.489 for distal and literal misclassification and of 0.671 for distal and metaphor misclassification). These results may indicate that distal metonymies partly overlap with literal senses, whereas metaphors have separate representations from literal senses. In addition, we showed that semantic extensions of the same type tend to overlap more than semantic extensions of different types: the two proximal metonymies have the greatest number of overlaps (ARI of 0.292), and proximal metonymy and distal metonymy overlap more (ARI of 0.423) than either of the metonymies and metaphor (ARI of 0.636 for proximal metonymy and metaphor and of 0.671 for distal metonymy and metaphor).

Our second finding that metaphors may partly overlap with metonymies in adjectives is in line with the results in Lopukhina et al. (2018). In our experiment, metaphors were overwhelmingly classified correctly, however, when they were misclassified, they were more frequently grouped with metonymies than with literal senses. This may be interpreted as indirect evidence for the existence of the “gray area” between metaphor and metonymy previously noted in theoretical research (Goossens, 1990; Dirven, 2003; Taylor, 2003; Ruiz de Mendoza Ibáñez and Galera Masegosa, 2011; Qian, 2016). Also, this finding can be considered as a testimony of a continuous and non-linear nature of polysemy: “neighboring” senses tend to overlap. Metaphors, which come “after” metonymies on the scale of closeness to the literal senses, overlap with metonymies and not with literal senses, whereas metonymies overlap both with the literal senses and metaphors.

Interestingly, metaphors were confused with proximal metonymies more than with distal metonymies. Although both distal metonymy and metaphor represent greater leaps from the literal sense than proximal metonymy, it does not seem to bring them together. The explanation may be that language speakers perceive different semantic extensions as steps in different directions. As a result, the greater the step, the farther one gets not only from the center (literal sense), but also from other semantic extensions. Consequently, the “distance” between proximal metonymy and metaphor may be less than the “distance” between distal metonymy and metaphor, resulting in their better discernibility from each other. Also, it is possible that proximal metonymy and metaphor have a greater collocational overlap which leads to their greater confusion. Certain adjectival noun phrases are ambiguous between proximal metonymy and metaphor: e.g., *trezvyj vzgljad “*sober look” can refer either to the look of a person who is not inebriated (proximal metonymy) or to a sober outlook on life (metaphor); *delikatnyj vopros “*delicate question” can refer either to a question asked by a tactful person (proximal metonymy) or to a sensitive issue (metaphor). This observation may become a hypothesis for further research.

Finally, our third finding confirmed the influence of the surrounding senses on the perception of semantic distinctions. This finding represents a methodological advancement which should be taken into account in designing non-timed sorting experiments. The fact that moderate semantic distinctions are perceived better in the presence of senses displaying more fine-grained distinctions but worse in the presence of senses displaying more coarse-grained distinctions, is of paramount importance. It corroborates the Graded Salience Hypothesis, developed in Giora (1997), according to which salient meanings receive priority in the psychological activation and semantic retrieval over less salient meanings in the process of language comprehension. It also likely reflects general cognitive mechanisms of human attention whereby salient stimuli receive an enhanced response at the cost of less salient stimuli which go unnoticed (Knudsen, 2007).

Potentially, the entire system of polysemy in words represents a continuum. The less related the senses are, the better they are identified as separate. We suppose that the mental representation of metaphor is not homogeneous either. For example, one might agree that a color metaphor, such as *golden hair*, is more related to the literal sense of *golden* (*golden ring*) than good quality metaphor *golden heart*, and that may indicate their different storage in the mental lexicon. In fact, our own data suggest differences in sense-relatedness between metaphors and hence their overlaps with literal senses. The qualitative analysis of individual baskets shows that personification metaphor like *umnye bomby “*smart bombs” was sometimes confused with the literal sense *umnyj chelovek* “smart person,” while synesthetic metaphor like *saxarnye rechi “*sugar.ADJ speeches” was never confused with the literal sense *saxarnye bulki “*sugar.ADJ buns.”

On the one hand, our study reveals certain limitations of relying on purely behavioral offline methods for making claims about the structure of the mental lexicon, especially in the light of how the speakers’ judgment can be affected by the linguistic surroundings. Without a supporting electrophysiological or neuroimaging experiment, our conclusions are tentative. On the other hand, the non-timed behavioral nature of the experiment has its strengths, which are not afforded by online methods. On the whole, it appears that the mental lexicon is a phenomenon that functions on more than one level. And we suppose that a conscious understanding of the lexicon structure can be better captured in a non-timed behavioral task. On close examination and deeper thinking, speakers may become aware of semantic patterns they are not mindful of when processing the language. However, their judgment is still indicative of perceived cognitive differences between semantically different phenomena, that are not as readily capturable by online methods.

On the whole, we find that experimental and theoretical approaches can inform and advance each other in meaningful ways. Experimental approaches can profit from the accumulated theoretical knowledge of polysemy by posing new questions that take into account the complexity of the object of study. Our experiment has shown that fine-grained classifications of semantic extensions are not purely theoretical conjectures, but do, to an extent, reflect the underlying intuitions of the speakers and, therefore, provide an insight into the potential structure of the mental lexicon. Likewise, theoretical methods can rely on experimental evidence to adjust the suggested classifications. We were able to demonstrate that semantic processing may be affected by context, and therefore, semantic distinctions among polysemes are not as firmly established as dictionaries or theoretical scholars might suggest, but are, to an extent, a matter of variation. Likewise, there are no strict semantic borders among the senses of a polysemous word, but the polysemes form a continuum with fuzzy boundaries, yet perceivably different distances among different semantic extensions.

We acknowledge that our study has two limitations. First, our stimuli were Russian adjectives and were limited by the peculiarities of the Russian adjectival polysemy system. Most stimuli used in the experiment were based on qualitative adjectives and are reproducible in English, cf. proximal metonymies *intelligent person - intelligent eyes* and *intelligent person - intelligent conversation*; temporal distal metonymy *hungry person - hungry years*; causal distal metonymy *sad person - sad news*. However, resultative metonymy, which is based on relative adjectives, seems to be peculiar to Russian and some other Slavic languages, while in English this meaning is expressed by nouns rather than adjectives: cf. *lead poisoning* (not ^∗^*leaden poisoning*), *milk allergy* (not ^∗^*milky allergy*). Second, our current research focused on different types of metonymy and did not include any gradation of sense-relatedness within metaphor. This limitation did not allow us to fully test the differences in mental storage between different kinds of metonymy and different kinds of metaphor. Therefore, we were unable to investigate the findings from Jager and Cleland (2015) who suggested that certain types of metaphors are semantically closer to the literal senses than certain types of metonymy, and might be stored together with the literal senses as opposed to separately stored metonymies.

Future research could head in several directions. First, we would check our results against other languages that possess a system of adjectives with similar metonymic and metaphorical extensions. Second, we would expand the experiment to include different types of metaphors, with different degrees of proximity to the literal sense. For example, the widespread and cognitively transparent conceptual metaphor MORE IS UP, such as *high pressure* or *high temperature*, may be perceived as less distinct from the literal sense (*high mountains*) than the more abstract temporal-spatial metaphor LATER IS HIGHER, as in *high summer* or *high season.* Thus, a follow-up behavioral study will control for fine-grained semantic differences within each of the semantic extensions, thus enhancing our understanding of the entire phenomenon of polysemy and its representation in the mental lexicon. Finally, previous experimental studies have demonstrated certain discrepancies in their results depending on the type of experimental paradigms and measures. It appears that behavioral experiments, such as reported in (Klein and Murphy, 2001, 2002; Klepousniotou and Baum, 2007; Klepousniotou et al., 2008; Lopukhina et al., 2018), tend to support separate storage for at least some types of polysemes, whereas neuroimaging and eye-tracking studies, with some exceptions (Yurchenko et al., 2020), more often suggest single storage for polysemes (Frisson and Pickering, 1999; Beretta et al., 2005; Frisson, 2009). A further inquiry into the neuronal representation of polysemy seems important, in order to clarify the source of the apparent discrepancy between the results of behavioral and neuroimaging studies into the storage and processing of polysemous words.

## Data Availability Statement

The datasets generated and analyzed for this study and other supplementary material can be found in the following GitHub repository: https://github.com/MariaZarifyan/Representation-of-different-types-of-adjectival-polysemy-in-the-mental-lexicon.

## Ethics Statement

The studies involving human participants were reviewed and approved by HSE Committee on Interuniversity Surveys and Ethical Assessment of Empirical Research. The patients/participants provided their written informed consent to participate in this study.

## Author Contributions

VA: conceptualization, methodology, supervision, writing – original draft, writing – review and editing, and investigation. AL: methodology, formal analysis, resources, software, and writing – review and editing. MZ: data curation, formal analysis, resources, software, visualization, and investigation. All authors contributed to the article and approved the submitted version.

## Conflict of Interest

The authors declare that the research was conducted in the absence of any commercial or financial relationships that could be construed as a potential conflict of interest.

## Publisher’s Note

All claims expressed in this article are solely those of the authors and do not necessarily represent those of their affiliated organizations, or those of the publisher, the editors and the reviewers. Any product that may be evaluated in this article, or claim that may be made by its manufacturer, is not guaranteed or endorsed by the publisher.

## Footnotes

1
https://toloka.yandex.com

2 We have not recruited participants based on their gender. The fact that in our experiment female participants are twice the number of male participants may be explained by the observation that women are more likely than men to volunteer (Freeman, 1997). Note that in many previous studies on polysemy the majority of the participants were women (e.g., Pylkkänen et al., 2006; Jager and Cleland, 2015; Weiland-Breckle and Schumacher, 2018).

3 All the experimental materials, data and code are available online: https://github.com/MariaZarifyan/Representation-of-different-types-of-adjectival-polysemy-in-the-mental-lexicon

4
www.ruscorpora.ru/en/

5
https://github.com/MariaZarifyan/Representation-of-different-types-of-adjectival-polysemy-in-the-mental-lexicon

6
http://sgs.ll-cl.org/

## References

[B1] Anashkina E. Ivanova O. (2016). The functioning mechanism of attributive metonymy in english fictional discourse. *Hum. Soc. Sci.* 9 2315–2327. 10.17516/1997-1370-2016-9-10-2315-2327

[B2] Apresjan J. D. (1974). Regular polysemy. *Linguistics* 142 5–32. 10.1515/ling.1974.12.142.5

[B3] Apresjan J. D. (1995). *Lexical Semantics. Selected Works.* Moscow: Jazyki russkoj kul’tury.

[B4] Apresjan J. D. Apresjan V. J. Babaeva E. E. Boguslavskaja O. J. Galaktionova I. V. (2014). *Active Dictionary of the Russian Language*. Moscow: Aktivnyj slovar’ russkogo jazyka. 1–2.

[B5] Apresjan J. D. (ed.) (2017). *Active Dictionary of Russian*, Vol. 3. Moscow: Jazyki russkoj kul’tury.

[B6] Arapinis A. (2015). Whole-for-part metonymy, classification, and grounding. *Linguist. Philos.* 38 1–29. 10.1007/s10988-014-9164-6

[B7] Barcelona A. (2003). “On the plausibility of claiming a metonymic motivation for conceptual metaphor,” in *Metaphor and Metonymy at the Crossroads: A Cognitive Perspective*, ed. Barcelona A. (Moscow: Jazyki slavjanskih kul’tur), 31–58. 10.1515/9783110894677.31

[B8] Beretta A. Fiorentino R. Poeppel D. (2005). The effects of homonymy and polysemy on lexical access: an MEG study. *Brain Res. Cogn. Brain Res.* 24 57–65. 10.1016/j.cogbrainres.2004.12.006 15922158

[B9] Birih A. (1995). *Metonimija v Sovremennom Russkom Jazyke [Metonymy in Modern Russian Language].* Bern: Peter Lang D. 10.3726/b12448

[B10] Copestake A. Briscoe T. (1995). Semi-productive polysemy and sense extension. *J. Seman.* 12 15–67. 10.1093/jos/12.1.15

[B11] Croft W. (2003). “The role of domains in the interpretation of metaphors and metonymies,” in *Metaphor and Metonymy in Comparison and Contrast. Ralf Pörings*, ed. Dirven R. (Berlin: Mouton de Gruyter), 161–206.

[B12] Cruse D. A. (1986). “Lexical semantics,” in *International Encyclopedia of the Social & Behavioral Sciences*, eds Smelser N. J. Baltes P. B. Wright J. D. (Amsterdam: Elsevier).

[B13] Dirven R. (1999). “Conversion as a conceptual metonymy of an event structure,” in *Metaphor and Metonymy in Comparison and Contrast*, eds Dirven R. Pörings R. (Berlin: Mouton de Gruyter), 275–287.

[B14] Dirven R. (2003). *Metaphor and Metonymy in Comparison and Contrast.* Berlin: Mouton de Gruyter. 10.1515/9783110219197

[B15] Falkum I. L. Vicente A. (eds) (2015). Polysemy: current perspectives and approaches. *Lingua* 157 1–16. 10.1016/j.lingua.2015.02.002

[B16] Fass D. (1997). *Processing Metonymy and Metaphor.* Oxford: Ablex Publishing.

[B17] Frazer J. G. (1923). *The Golden Bough: A Study in Magic and Religion.* Berlin: Springer. 10.1007/978-1-349-00400-3

[B18] Freeman R. B. (1997). Working for nothing: the supply of volunteer labor. *J. Labor Econ.* 15 S140–S166. 10.1086/209859

[B19] Freud S. (1900). “The interpretation of dreams,” in *The Standard Edition of the Complete Works of Sigmund Freud, 4, 5*, ed. Strachey J. (London: Hogarth Press).

[B20] Frisson S. (2009). Semantic underspecification in language processing. *Lang. Linguist. Compass* 3 111–127. 10.1111/j.1749-818X.2008.00104.x

[B21] Frisson S. Pickering M. J. (1999). The processing of metonymy: evidence from eye movements. *J. Exp. Psychol. Learn. Mem. Cogn.* 25 1366–1383. 10.1037/0278-7393.25.6.1366 10605827

[B22] Giora R. (1997). Understanding figurative and literal language: the graded salience hypothesis. *Cogn. Linguist.* 8 183–206. 10.1515/cogl.1997.8.3.183 31158291

[B23] Goossens L. (1990). Metaphtonymy: the interaction of metaphor and metonymy in expressions for linguistic action. *Cogn. Linguist.* 1 323–342. 10.1515/cogl.1990.1.3.323

[B24] Goossens L. (1999). “Metonymic bridges in modal shifts,” in *Metonymy in Language and Thought*, eds Panther K.-U. Radden G. (London: Hogarth Press), 193–210. 10.1075/hcp.4.11goo

[B25] Goossens L. (2003). “Les opérateurs-n en Grammaire Fonctionelle, les verbes modaux de l’anglais, et ‘grounding’,” in *Développements Récents en Grammaire Fonctionelle*, ed. Jadir M. (Mohammedia: Publications de la Faculté de Lettres et des Sciences Humaines, Série Colloques 16, Université Hassan II), 121–133.

[B26] Hubert L. Arabie P. (1985). Comparing partitions. *J. Classif.* 2 193–218. 10.1007/BF01908075

[B27] Jager B. Cleland A. A. (2015). Connecting the research fields of lexical ambiguity and figures of speech. *Ment. Lexicon* 10 133–151. 10.1075/ml.10.1.05jag 33486653

[B28] Jakobson R. Halle M. (1956). “Two aspects of language and two types of aphasic disturbances,” in *Fundamentals of Language* 2nd Edn. (Mouton: The Hague), 69–96.

[B29] Klein D. E. Murphy G. L. (2001). The representation of polysemous words. *J. Mem. Lang.* 45 259–282. 10.1006/jmla.2001.2779

[B30] Klein D. E. Murphy G. L. (2002). Paper has been my ruin: conceptual relations of polysemous senses. *J. Mem. Lang.* 47 548–570. 10.1016/S0749-596X(02)00020-7

[B31] Klepousniotou E. (2002). The processing of lexical ambiguity: homonymy and polysemy in the mental lexicon. *Brain Lang.* 81 205–223. 10.1006/brln.2001.2518 12081393

[B32] Klepousniotou E. Baum S. (2007). Disambiguating the ambiguity advantage effect in word recognition: an advantage for polysemous but not homonymous words. *J. Neurolinguist.* 20 1–24. 10.1016/j.jneuroling.2006.02.001

[B33] Klepousniotou E. Pike B. Steinhauer K. Gracco V. (2012). Not all ambiguous words are created equal: an EEG investigation of homonymy and polysemy. *Brain Lang.* 123 11–21. 10.1016/j.bandl.2012.06.007 22819308

[B34] Klepousniotou E. Titone D. Romero C. (2008). Making sense of word senses: the comprehension of polysemy depends on sense overlap. *J. Exp. Psychol.* 34 1534–1543. 10.1037/a0013012 18980412

[B35] Knudsen E. I (2007). Fundamental components of attention. *Annu. Rev. Neurosci.* 30 57–78. 10.1146/annurev.neuro.30.051606.094256 17417935

[B36] Kövecses Z. (1986). *Metaphors of Anger, Pride and Love: A Lexical Approach to the Structure of Concepts.* Amsterdam: John Benjamins. 10.1075/pb.vii.8

[B37] Kövecses Z. (1990). *Emotion Concepts.* New York, NY: Springer. 10.1007/978-1-4612-3312-1

[B38] Kövecses Z. (2002). Cognitive-linguistic comments on metaphor identification. *Lang. Literature* 11 74–78. 10.1177/096394700201100107

[B39] Kövecses Z. (2010). *Metaphor. A Practical Introduction*. 2nd Edn. New York: Oxford University Press.

[B40] Kövecses Z. (2013). The metaphor–metonymy relationship: correlation metaphors are based on metonymy. *Metaphor Symb.* 28 75–88. 10.1080/10926488.2013.768498

[B41] Kustova G. I. (1998). Proizvodnye znachenija s eksperientsialnoj sostavljaushchej. *Semiotika Inform.* 36 19–40.

[B42] Kustova G. I. (2004). *Tipy Proizvodnykh Znachenij i Mekhanizmy Jazykovogo Rasshirenija.* Moscow: Jazyki slavjanskoj kultury.

[B43] Lacan J. (1957). *Formations of the Unconscious, The Seminar of Jacques Lacan, Book V.* London: Polity Press.

[B44] Lakoff G. Johnson M. (1980). Conceptual metaphor in everyday language. *J. Philos.* 77:453. 10.2307/2025464

[B45] Lehrer A. (1990). Polysemy, conventionality, and the structure of the lexicon. *Cogn. Linguist.* 1 207–246. 10.1515/cogl.1990.1.2.207

[B46] Lopukhina A. Laurinavichyute A. Lopukhin K. Dragoy O. (2018). The mental representation of polysemy across word classes. *Front. Psychol.* 9:192. 10.3389/fpsyg.2018.00192 29515502 PMC5826358

[B47] MacGregor L. J. Bouwsema J. Klepousniotou E. (2015). Sustained meaning activation for polysemous but not homonymous words: evidence from EEG. *Neuropsychologia* 68 126–138. 10.1016/j.neuropsychologia.2015.01.008 25576909

[B48] Nunberg G. (1979). The non-uniqueness of semantic solutions: polysemy. *Linguist. Philos.* 3 143–184. 10.1007/BF00126509

[B49] Paducheva E. V. (2004). *Dinamicheskie Modeli v Semantike Leksiki.* Moscow: Jazyki slavjanskoj kultury.

[B50] Panther K. Thornburg L. L. (2004). *The Role of Conceptual Metonymy in Meaning Construction.* Amsterdam: John Benjamins Publishing.

[B51] Panther K.-U. Radden G. (1999). *Metonymy in Language and Thought.* Amsterdam: John Benjamins Publishing. 10.1075/hcp.4

[B52] Pedregosa F. Varoquaux G. Gramfort A. Michel V. Thirion B. Grisel O. (2011). Scikit-learn: machine learning in python. *J. Mach. Learn. Res.* 12 2825–2830.

[B53] Peirsman Y. Geeraerts D. (2006). Metonymy as a prototypical category. *Cogn. Linguist.* 17 269–316. 10.1515/COG.2006.007

[B54] Pustejovsky J. (1995). *The Generative Lexicon.* Cambridge, MA: MIT Press.

[B55] Pylkkänen L. Llinás R. Murphy G. L. (2006). The representation of polysemy: MEG evidence. *J. Cogn. Neurosci.* 18 97–109. 10.1162/089892906775250003 16417686 PMC1351340

[B56] Qian L. (2016). Metonymic-based metaphor—a case study on the cognitive interpretation of “heart” in english and Chinese. *High. Educ. Stud.* 6:131. 10.5539/hes.v6n4p131

[B57] Radden G. Kövecses Z. (1999). “Towards a theory of metonymy,” in *Metonymy in Language and Thought*, eds Panther K.-U. Radden G. (Philadelphia: John Benjamins), 17–60.

[B58] R Core Team (2016). *R: A Language and Environment for Statistical Computing.* Vienna: R Foundation for Statistical Computing.

[B59] Radden G. (2000). “How metonymic are metaphors?,” in *Metaphor and Metonymy at the Crossroads: A Cognitive Perspective*, ed. Barcelona A. (Moscow: Mouton de Gruyter), 93–108. 10.1515/9783110894677.93

[B60] Radden G. Kövecses Z. (1999). “Towards a theory of metonymy,” in *Metonymy in Language and Thought*, eds Panther K.-U. Radden G. (Amsterdam: John Benjamins Publishing), 17–59. 10.1075/hcp.4.03rad

[B61] Rakhilina E. Karpova O. Reznikova T. (2009). *Modeli Semantièeskoj Derivacii Mnogoznaènyh Kaèestvennyh Prilagatel’nyh: Metafora, Metonimija i Ih Vzaimodejstvie [Semantic-Derivational Models of Polysemous Adjectives: Metaphor, Metonymy, and Their Interaction], Komp’juternaja Lingvistika i Intellektual’nye Texnologii: Po Materialam Ežegodnoj Meždunarodnoj Konferencii “Dialog’2009”.* Moscow: RGGU, 420?–?425.

[B62] Reznikova T. Rakhilina E. Karpova O. Kyuseva M. Ryzhova D. Arkhangelskiy T. (2013). “Polysemy patterns in russian adjectives and adverbs,” in *Current Studies in Slavic Linguistics*, ed. Chahine I. K. (Amsterdam: John Benjamins Publishing). 10.1075/slcs.146.18rez

[B63] Reznikova T. I. Rakhilina E. Bonch-Osmolovskaya A. (2012). Towards a typology of pain predicates. *Linguistics* 50:15. 10.1515/ling-2012-0015

[B64] Rice S. (1992). “Polysemy and lexical representation: the case of three English prepositions,” in *Proceedings of the Fourteenth Annual Conference of the Cognitive Science Society*, (Lawrence, NJ: Lawrence Erlbaum), 89–94.

[B65] Rodd J. Gaskell G. Marslen-Wilson W. (2002). Making sense of semantic ambiguity: semantic competition in lexical access. *J. Mem. Lang.* 46 245–266. 10.1006/jmla.2001.2810

[B66] Ruiz de Mendoza Ibáñez F. J. Galera Masegosa A. (2011). Going beyond metaphtonymy: metaphoric and metonymic complexes in phrasal verb interpretation. *Lang. Value* 3 1–29. 10.6035/LanguageV.2011.3.2

[B67] Sandakova M. V. (2010). “Adjektivnaja metonimia v leksikograficheskom aspekte,” in *Vestnik Nizhegorodskogo Universiteta Im*, ed. Lobachevskogo N. I. (Lawrence, NJ: Lawrence Erlbaum), 712–715.

[B68] Sandakova M. V. (2015). “Metonimia kak osnova slovoobrazovatelnoj motivatsii (na materiale prilagatelnogo),” in *Vestnik Nizhegorodskogo Universiteta Im*, ed. Lobachevskogo N. I. (Lawrence, NJ: Lawrence Erlbaum), 541–546.

[B69] Seto K. (1999). “Distinguishing metonymy from synecdoche,” in *Metonymy in Language and Thought*, eds Panther K.-U. Radden G. (Lawrence, NJ: Lawrence Erlbaum), 91–120. 10.1075/hcp.4.06set

[B70] Tamminen J. Cleland A. A. Quinlan P. T. Gaskell M. G. (2006). “Processing semantic ambiguity: different loci for meanings and senses,” in *Proceedings of the Twenty-Eighth Annual Conference of the Cognitive Science Society*, (Mahwah, NJ: Lawrence Erlbaum Associates), 2222–2227.

[B71] Taylor J. (2003). Polysemy’s paradoxes. *Lang. Sci.* 25 637–655. 10.1016/S0388-0001(03)00031-7

[B72] Vosshagen C. (1999). “Opposition as a metonymic principle,” in *Metonymy in Language and Thought*, eds Panther K.-U. Radden G. (Amsterdam: John Benjamins Publishing Company), 289–308. 10.1075/hcp.4.17vos

[B73] Warren B. (2006). *Referential Metonymy.* Lund: Almqvist & Wiksell International.

[B74] Warren B. (2009). “An alternative account of the interpretation of referential metonymy and metaphor,” in *Metaphor and Metonymy in Comparison and Contrast*, eds Dirven R. Pörings R. (Berlin: De Gruyter Mouton), 113–132.

[B75] Weiland-Breckle H. Schumacher P. B. (2018). A direct comparison of metonymic and metaphoric relations in adjective–noun pairs. *Acta Linguist. Acad.* 65 443–472. 10.1556/2062.2018.65.2-3.8

[B76] Yurchenko A. Lopukhina A. Dragoy O. (2020). Metaphor is between metonymy and homonymy: evidence from event-related potentials. *Front. Psychol.* 11:2113. 10.3389/fpsyg.2020.02113 32982863 PMC7490628

